# Deep carbon storage potential of buried floodplain soils

**DOI:** 10.1038/s41598-017-06494-4

**Published:** 2017-08-15

**Authors:** Amanda H. D’Elia, Garrett C. Liles, Joshua H. Viers, David R. Smart

**Affiliations:** 1 Department of Viticulture & Enology, University of California, One Shields Avenue, Davis, CA 95616 USA; 2 School of Engineering University of California, Merced, 5200N. Lake Road, Merced, CA 95343 USA; 3 College of Agriculture California State University, Chico 400 West First Street, Chico, CA 95929 USA

## Abstract

Soils account for the largest terrestrial pool of carbon and have the potential for even greater quantities of carbon sequestration. Typical soil carbon (C) stocks used in global carbon models only account for the upper 1 meter of soil. Previously unaccounted for deep carbon pools (>1 m) were generally considered to provide a negligible input to total C contents and represent less dynamic C pools. Here we assess deep soil C pools associated with an alluvial floodplain ecosystem transitioning from agricultural production to restoration of native vegetation. We analyzed the soil organic carbon (SOC) concentrations of 87 surface soil samples (0–15 cm) and 23 subsurface boreholes (0–3 m). We evaluated the quantitative importance of the burial process in the sequestration of subsurface C and found our subsurface soils (0–3 m) contained considerably more C than typical C stocks of 0–1 m. This deep unaccounted soil C could have considerable implications for global C accounting. We compared differences in surface soil C related to vegetation and land use history and determined that flooding restoration could promote greater C accumulation in surface soils. We conclude deep floodplain soils may store substantial quantities of C and floodplain restoration should promote active C sequestration.

## Introduction

Soils are the largest carbon reservoir of the terrestrial biosphere containing approximately 2,500 Pg of carbon and have the potential to sequester large quantities of C^1, 2^. Buried soils have recently been suggested as important sources of unaccounted SOC^3^. Buried soils occur in various landscapes globally ranging from volcanic settings to aeolian deposits as well as alluvial floodplain systems. Despite the potential for substantial buried C stocks in a variety of globally widespread environments, current SOC inventories are constrained to 1 m^4, 5^. Typical soil chronosequences exhibit an exponential decrease in SOC with depth that is currently used as the basis for 1 m soil carbon models^6, 7^. SOC stocks beyond this 1 m^3^ depth have previously been considered to provide a negligible input to total C pools representing less dynamic C pools^8^. However, various burial processes can effectively store large stocks of carbon at depths significantly greater than 1 m^3^. In particular, burial due to flooding can result in prolonged saturation and sediment deposition over organically rich surface soils promoting both SOC stabilization and sequestration due to decreased microbial activity^9–12^.

Floodplains cover 0.5–1% of the global land area but have been suggested to account for a range of 0.5–8% of global SOC storage^11^. River networks contain significant portions of terrestrial C with greatest retention occurring in floodplain riparian ecosystems^13–16^. In particular broad and unconfined valleys tend to decrease transport energy, which promotes deposition and storage of organic materials^11, 17^. The above kinds of systems are prolific in delta floodplains where aggradation is favored and there is increased lateral and vertical hydrologic connectivity, factors known to contribute to increased SOC storage^11, 13, 18^. Ecological degradation through removal of riparian forests for agricultural production and hydrologic disconnection resulting from channelization and levee construction has effectively limited these systems from realizing their natural C storage potential^18, 19^. Dam construction has also created legacy effects by reducing sediment supplies and limiting deposition potential, which inherently limits downstream C storage potential^20^.

Located along the Cosumnes River in northern California, the last undammed major river flowing out of the western Sierra Nevada Mountains, the Cosumnes floodplain contains thick alluvial deposits known to be thousands of years old resulting from the uplift and subsequent erosion of the Sierra Nevada batholith^21^. In the last 150 years, anthropogenic alteration of global floodplain areas of which the lower Cosumnes River is an example, has resulted in significant limitation of functional floodplain area^11^. Prior to disturbance, the lower Cosumnes River was an anastomosing river that contained multiple channels, seasonal marshes and perennial floodplain lakes^22^. Flooding has mostly been restricted within a single channel for nearly 100 years. This effectively disconnected the river from its floodplain resulting in a considerable decline in biodiversity. In this report, we examined the SOC stocks of the Cosumnes California delta floodplain to assess the importance of buried soils in deep unaccounted for SOC and to evaluate the C sequestration potential of floodplain restoration.

## Results

### Subsurface Soils

**Figure 1:**
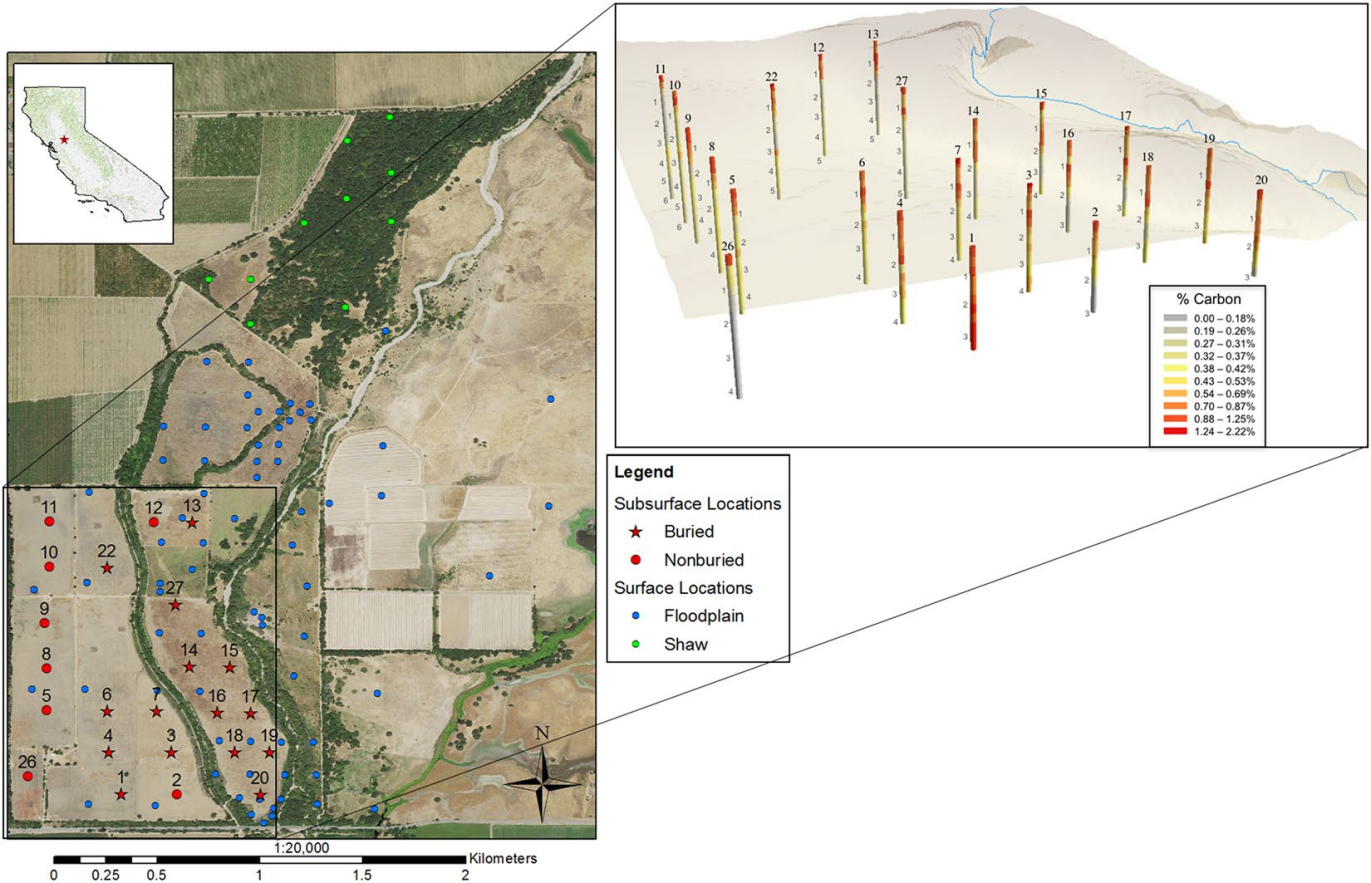
Map of the field site with (i) subsurface borehole sites as red stars (with a buried horizon) and red circles (without a buried horizon) and (ii) surface sample sites in dark blue (floodplain ecosystem) and light blue (Shaw forest ecosystem). The offset map shows the subsurface carbon distribution at depth where gray vertical numbers indicate depth (m), red indicates higher carbon and gray indicates lower carbon. Both maps were created with ArcGIS version 10.4.1 (https://www.arcgis.com/features/index.html).

**Figure 2:**
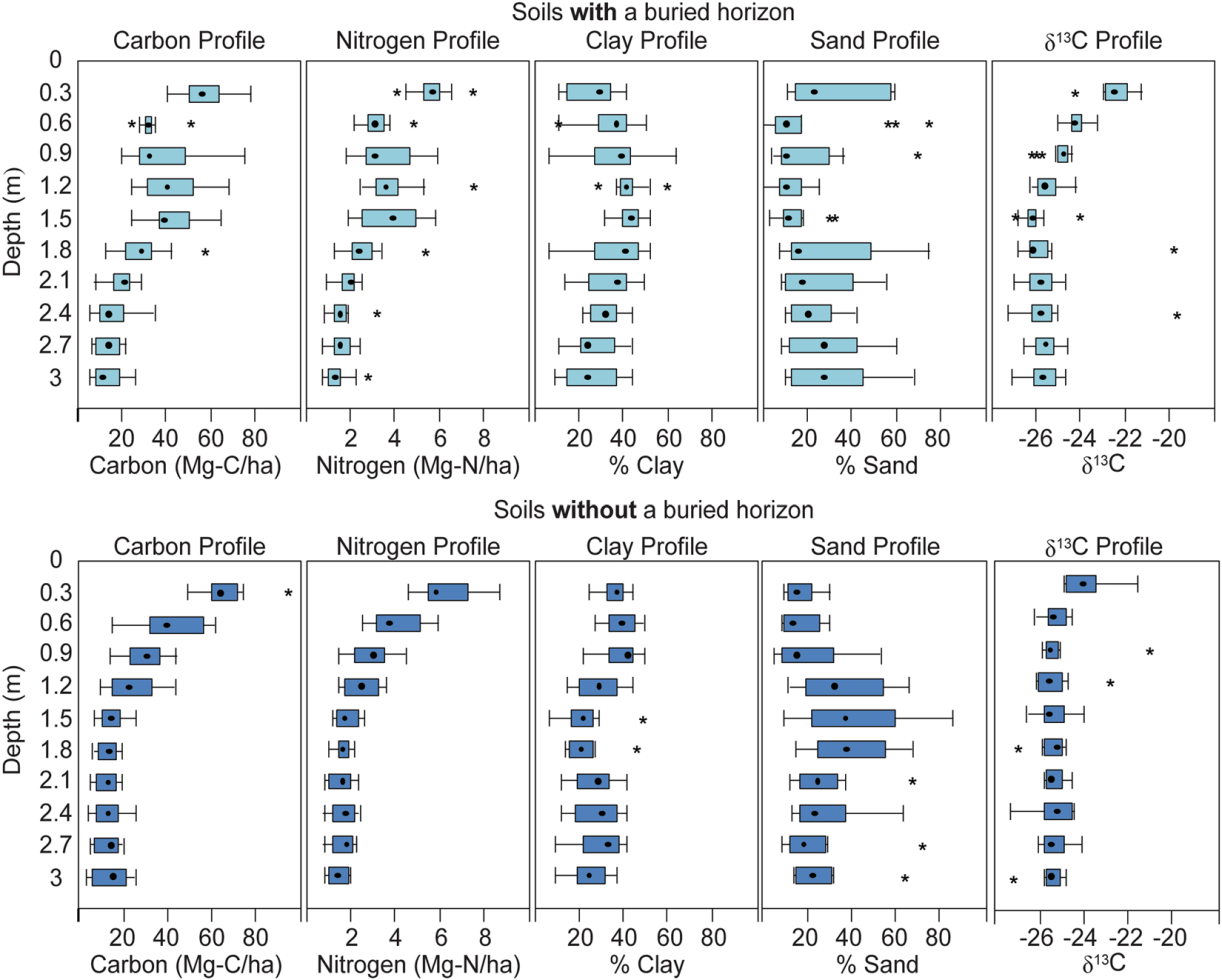
Depth profiles of C, N, clay, and sand contents, and δ^13^C for the soils with a buried horizon and those without. Asterisks indicate outliers, whiskers indicate max and min values, boxes represent the 25% and 75% quantiles, and the bolded dot is the median.

**Figure 3:**
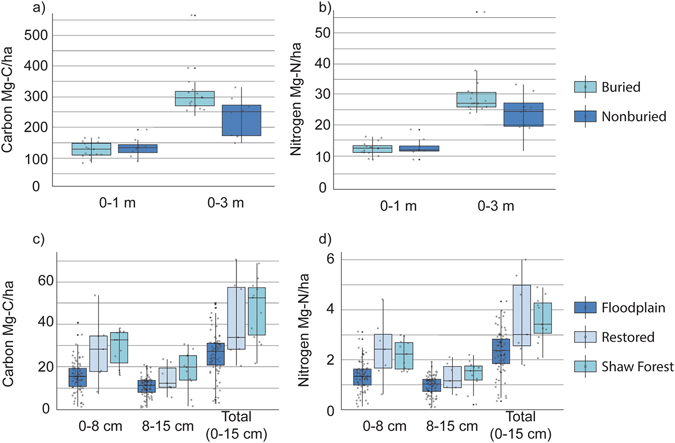
Box and whisker plots indicate the mean total C and N in Mg/ha for subsurface and surface samples. (**a**,**b**) Represent mean sums from 0–1 m and 0–3 m for subsurface soils with a buried horizon and subsurface soils without a buried horizon. (**c**,**d**) Represent mean sums from 0–8 cm, 8–15 cm and 0–15 cm for surface soils found in the Shaw forest, the floodplain and previously restored sites. Asterisks indicate outliers, whiskers indicate max and min values, the boxes represent the 25% and 75% quantiles and the hollow points are the discrete data points used to create the box plot.

While examining our 23 subsurface soil boreholes, we found a buried horizon in 15 of 23 boreholes (Fig. 1). The buried horizons occurred at approximately 1–2 m depth (Fig. 2) and likely represent the remnants of the functioning floodplain system prior to disturbance containing significant amounts of the total SOC stored in these soils. These horizons were noticeably darker in color, a signal of increased SOC content, and were characterized as a profile segment below 30 cm that did not have significantly different SOC content than the current soil surface (p > 0.05). They also showed an increase in C from the overlying segment of greater than 50% (see Supplementary Table S1) and thus fit the definition of a buried horizon outlined by the NRCS^23^. Total SOC stocks for subsurface samples containing a buried horizon were significantly greater than those boreholes without them. This translated into an average increase of approximately 34% more total C/ha at 0–3 m for soils with a buried horizon (Fig. 3, see Supplementary Table S2). In comparing the SOC of all cores (0–3 m) to the typical depth of SOC stocks (0–1 m), we found significantly more SOC when depths greater than 1 m were included (Fig. 3, see Supplementary Table S2). Without differentiating between soils with and without a buried horizon, SOC in soils quantified from 0–3 m were on average more than double the SOC from 0–1 m (Fig. 3, see Supplementary Table S2). Differences between soils with and without the buried horizon were not seen at 0–1 m depth because the top of the buried horizon generally started at 1 m (Fig. 2).

In comparing total SOC from 0–1 m of these soils with typical California SOC stocks from 0–1 m across various land uses, these soils with 129 Mg-C/ha fall close to the mean (133 Mg-C/ha) and slightly below what is usually found in cropland systems (188 Mg-C/ha)^24^. However, when taken from 0–3 m, our SOC stocks (286 Mg-C/ha) were comparable to or exceed what is seen in forests, pastures and wetlands from 0–1 m (220, 260, 215 Mg-C/ha respectively)^24^, and well above typical cropland stocks from 0–1 m. The fact that the buried horizon was not continuously present in all boreholes at our field site demonstrates the high variability inherent in multichannel floodplain systems at the landscape scale. However, in studying maps created before extensive human disturbance began, most boreholes without a buried horizon occurred along what was once the original Cosumnes River channel (see Supplementary Figure S1). Because these sites are located in former river channel, burial processes with extensive sediment deposition are not expected. Thus, no buried horizons should occur in this area. This indicated that while accounting for variability may be important in estimating field scale SOC stocks, this particular field site has a somewhat continuous layer of buried SOC.

The buried horizon at this site had increased clay content and decreased sand content, which demonstrates the general relationship between clay sized particles and SOC storage (Fig. 2, see Supplementary Figure S2). We also found increased nitrogen (N) retention at this buried horizon indicating that decomposition of organic matter became restricted (Fig. 3). We found a second buried horizon at approximately 3 m depth in 7 out of 23 boreholes providing further evidence of past burial processes at the floodplain site (see Supplementary Table S3).

The isotopic data collected does not show evidence of decomposition in these soils as is seen in the natural abundance of ^13^C (δ^13^C) in soil profiles (Fig. 2). Soil typically becomes more enriched in δ^13^C with depth as a consequence of decomposition^26^, but enrichment was not seen in our deep boreholes. In fact, the δ^13^C signature indicated ^13^C content became more depleted with depth most likely because this δ^13^C profile represented a shift in the ecological factors that affect organic matter δ^13^C signatures^26^. These include water availability, topography, salinity, and, most likely, shifts in vegetation^26^. It is possible that a shift in vegetation C inputs, from native C3 vegetation with δ^13^C at −33 to −24‰, to corn with δ^13^C depletions of −16 to −10‰, a C4 agricultural crop and the predominant crop grown at the leveed areas of the field site contributed to the overall shift in the δ^13^C.

### Surface Soils

We found a significant difference in surface SOC when comparing the three primary soil land use histories (Fig. 3). SOC in the surface soil of the Shaw Forest (0–15), an undisturbed old growth oak gallery forest, was significantly greater than the surface soil of the rest of the floodplain which has been disturbed by agricultural cultivation. This was an expected observation for the two ecosystem components as mature forest soils typically have higher SOC^27^. In comparing undisturbed forest SOC with SOC of soils found approximately 5 km south of our restoration area on parts of the Cosumnes floodplain undergoing restoration over 10 years ago, there was no significant difference in surface SOC content (Fig. 3). This indicated seasonal flooding within these floodplain ecosystems can support rapid accumulation of SOC to pre-disturbance levels over relatively short time-intervals. This is strong evidence that restoration of floodplains can increase C sequestration. The buried C-rich horizons found in our subsurface soil boreholes provided additional evidence for active C sequestration that might occur within the floodplain.

## Discussion

We found a substantial amount of C in our deep soil boreholes that is not currently accounted for in SOC stock assessments (to 1 m). There are two factors in global SOC stock estimates and C models that do not adequately represent these and other buried soils. The first is the exclusion of soil below 1 m, which is rooted in our understanding of SOC cycling processes primarily as they relate to agriculture in the upper 30 cm or the rooting zone^25^. As soil science has evolved to incorporate biogeochemical cycling concepts of natural systems, the definition of soil will need to extend below the 2 m lower limit defined by the USDA Soil Taxonomy^23^. The second factor is the misrepresentation of soils as homogenous at depth in regards to SOC. Not all soils show typical exponential decay of SOC with depth. Our floodplain soils and many other soils, including other types of buried soils and SOC-rich soils found in wetlands, marshes and peatlands fall into this category. Incorporation of these highly variable and heterogeneous soils into current SOC stocks and global C models will be challenging, but it is clear from this study they contain significant amounts of stable SOC currently ignored in terrestrial C stock assessments. Thus, understanding of SOC may need to extend beyond the current definitions used in our models and estimates.

To highlight the magnitude of the difference in SOC between soils taken from 0–1 m and 0–3 m we completed a first-order approximation of global SOC stocks using our average SOC values from 0–1 m and 0–3 m (128 ± 5.72 Mg-C/ha and 286 ± 17.2 Mg-C/ha respectively) and the estimates of floodplain global land cover from Sutfin *et al*.^11^ (0.8 × 106 to 2 × 106 km^2^)^11^. Our estimations of total SOC for floodplains from 0–3 m were 25.6–57.2 Pg-C compared to just 10.2–25.6 Pg-C from 0–1 m (see Supplementary Table S4). Thus, floodplain soils could have an additional 12.6 to 31.6 Pg of SOC when calculated to 3 m depth (see Supplementary Table S4). First-order approximations are highly uncertain given then inherent margins of error when up-scaling any field scale data to a global scale. Because our C stocks were calculated using estimates of bulk density and SOM these margins are considerable. This approximation also assumes that all floodplains have the same environmental factors such as topography, geography, climate, vegetation, depth to groundwater and soil type which is known to be false. More refined approximations would require better use of remote sensing to more precisely calculate global floodplain area, more sophisticated calculations incorporating other floodplain data sets that represent different environmental factors and most likely modeling. However, these rough estimates demonstrate not only the influence floodplain soils have globally, but also the impact of using greater depths for floodplain SOC stocks.

As seen in the buried horizon at 1–2 m depth (Fig. 2), long-term storage of C in these soils requires the likely re-establishment of the inundation process. The overall objective of restoration is to hydrologically restore as much of the historic multichannel system and associated vegetation as possible. Following removal of levees in summer 2013 and implementation of larger levee setbacks at the north end of our field site we were successful in the important geomorphic process of multi-pathway flooding. The reconnection of river and floodplain has resulted in more regular and widespread flooding which has been shown to help reestablishment of riparian habitat and can result in large sediment deposition depending on the flood magnitude^22, 28^. For instance a flood on the Lower Cosumnes Floodplain of magnitude 206 m^3^s^−1^ (7274 cfs) deposited 943 ± 63 m^3^ of sediment in February 2015^29^. Hydrologic and vegetative restoration has shown in other floodplain systems to increase net C storage^30, 31^. Our surface soils showed that restoration of seasonal flooding can support rapid SOC accumulation to pre-disturbance levels and thus we expect SOC accumulation following restoration to occur over a relatively short time interval. We also expect C will be sequestered fairly long-term with C-enriched buried horizons as strong evidence. One caveat to sequestration is the biogeochemical cycling that occurs with groundwater fluctuation. Saturated conditions generally decrease metabolism of SOC by micorbes^12^. However, flooding events can create a sudden rise and fall in the water table creating moist conditions which are ideal for SOC metabolism^32^. Because the site is within close proximity to the main river channel, some microbial degradation of the SOC stocks nearest the channel may be expected with seasonal fluctuations in the water table. The rate of the degradation would be influenced by many other environmental factors. In general, this may put a lower depth limit on the amount of C that can be sequestered. Although the variability of C sequestered across floodplains can be high, the frequency of a C-enriched buried horizon at 1 m depth was also high. Our expectations are to provide a significant ecosystem service associated with riparian floodplain restoration.

The accumulation of buried SOC seen in these soils provides evidence of the beneficial impacts of functioning floodplain ecosystems. With increasing environmental pressure to offset atmospheric C emissions, new and innovative approaches are needed. Our findings in subsurface SOC stocks also highlight some limitations in current SOC assessments. It’s been suggested that large concentrations of terrestrial C that are not yet accounted for in C models can be found in the eroded materials accumulated at the toe-slopes of large uplifted regions^13^, but the general hypothesis lacks ground verification. The current depth limitation to SOC assessment may be drastically overlooking C accumulation in a variety of buried soils. As can be seen in our landscape-scale evaluation, these soils contain substantial subsurface SOC stocks currently unaccounted for in standard assessments. Better understanding of the scope and scale of delta floodplain soils and their inclusion in global C models could have a significant impact on current C budgets.

## Methods

### Soil Sampling and Preparation

Two sets of soil samples were taken over 350 ha corresponding to surface and subsurface carbon reservoirs. The surface samples were taken with a hand auger at 87 sites at depths representing the primary rooting zone of annual herbs (to 15 cm depth). The sampling structure overlapped with monument plots, for long-term monitoring along with additional dispersed sampling locations (Fig. 1). Subsurface samples (from 30 to 300 or 600 cm depth) were taken by hand auger at 23 sites across the restoration area by a contractor and were extracted in 30 cm increments. Sites were chosen across the floodplain in a regular grid pattern (Fig. 1). Depths for these boreholes varied depending upon water table encounter, and all samples were recovered above the water table. Soil from these samples were air dried for 48 hours, then oven dried at 60 °C for 48 hours and passed through a 2 mm sieve before conducting further analyses. No coarse fragments (>0.02 diameter) were present in these samples.

### C, N, δ^13^C and δ^15^N Analysis

A total of 553 subsurface and surface soil samples were analyzed for total carbon and total nitrogen as well as for δ^13^C and δ^15^N at the UC Davis Stable Isotope Facility (SIF) using a PDZ Europa ANCA-GSL elemental analyzer interfaced to a PDZ Europa 20–20 isotope ratio mass spectrometer (Sercon Ltd., Cheshire, UK). Samples were combusted at 1000 °C with a chromium oxide and silvered copper oxide packed reactor. After combustion, oxides were removed in a reduction reactor (reduced copper at 650 °C). The helium carrier gas then flowed through a water trap (magnesium perchlorate) and an optional CO_2_ trap (for N-only analyses). N_2_ and CO_2_ were separated on a Carbosieve GC column (65 °C, 65 mL/min) before entering the IRMS. A subset of samples with pH above 8 underwent an acid fumigation using 12 N HCl and were sent a second time to the UC Davis SIF for total carbon, total nitrogen, δ^13^C and δ^15^N analysis in order to analyze the amount of inorganic carbon present^33^. Inorganic carbon was found to be negligible, which allowed us to assume total carbon represented organic carbon content. Soil samples were also analyzed for pH using a 1:1 soil/DI water solution and a LAQUA F-71 pH meter (Horiba Ltd., Kyoto Japan)^34^.

### Other Soil Analysis

Particle size distribution was measured by hydrometer method for all soils^35^. Because we could not accurately measure bulk density, bulk soil density was estimated for all samples using the equations of Supplementary Tables S5 and S6 (available) and using the measured soil particle size distributions and estimations of organic matter^36^. While these calculations have inherent uncertainties, they are more robust than utilizing a single average bulk density for stock calculations. Soil organic matter (SOM) was estimated by multiplying measured total C values by 1.724 (the van Bemmelen factor)^37^ which also contains inherent uncertainties. Soil C stocks (Mg-C/ha) were calculated for each depth increment (*C*_*i*_) using equation (1) where *D*_*b*_ is bulk density (g/cm^3^), *z*_*i*_ is depth (m) and *C*_*w*_ is the

1 $${C}_{i}={D}_{b}\cdot {z}_{i}\cdot {C}_{w}\cdot \mathrm{10,000}$$

measured carbon concentration (% w/w). Because both SOM and bulk density were calculated rather than measured our SOC stocks contain inherent uncertainty. All statistical analyses were done in R 3.1.2^38^ and all values are considered significant (p < 0.05) unless otherwise specified.

### Data Availability

The datasets generated during and/or analyzed during the current study are available from the corresponding author on reasonable request.

## Electronic supplementary material

Supplementary Information
