# Supplementary material for: Deep carbon storage potential of buried floodplain soils
Source: Sci Rep. 2017 Aug 15;7:8181. doi: 10.1038/s41598-017-06494-4 (PMC5557967; doi:10.1038/s41598-017-06494-4)
Supplement: Supplementary file 1 — Supplementary Information [file 41598_2017_6494_MOESM1_ESM.pdf]

Supplementary Information

Deep carbon storage potential of buried floodplain soils

Authors:

Amanda D'Elia[1]
Garrett C. Liles [1†]
Joshua H. Viers[2]
David R. Smart[1*]

[1]Department of Viticulture & Enology
University of California
One Shields Avenue
Davis CA 95616

[2]School of Engineering
University of California, Merced
5200 N. Lake Road
Merced CA 95343

[†]College of Agriculture
California State University, Chico
400 West First Street
Chico CA 95929

***Corresponding Author:** Dr. David R. Smart
Department of Viticulture & Enology
University of California
One Shields Avenue
Davis, CA 95616
drsmart@ucdavis.edu
93-759-5712 (phone)
752-0382 (facsimile)

Supplementary Table S1. Buried soil horizon C contents and depths (bolded) compared to the overlying horizon (unbolded) including the percent increase in C at the buried horizon.

Borehole #	Depth (cm)	%C	% Increase
1	120-150	0.74	68
	**150-180**	**1.24**	
3	60-90	0.68	84
	**90-120**	**1.26**	
4	120-150	0.89	57
	**150-180**	**1.39**	
6	30-60	0.86	100
	**60-90**	**1.71**	
7	60-90	0.86	110
	**90-120**	**1.80**	
13	30-60	0.75	113
	**60-90**	**1.60**	
14	60-90	0.60	105
	**120-150**	**1.24**	
15	90-120	0.72	71
	**120-150**	**1.22**	
16	60-90	0.42	244
	**90-120**	**1.43**	
17	90-120	0.56	182
	**120-150**	**1.56**	
18	90-120	0.89	50
	**120-150**	**1.33**	
19	90-120	0.72	113
	**120-150**	**1.54**	
20	30-60	0.73	52
	**90-120**	**1.11**	
22	30-60	0.76	63
	**60-90**	**1.24**	
27	60-90	0.62	101
	**90-120**	**1.26**	

Supplementary Table S2. Measured total C and N contents of the Cosumnes subsurface soils for 0-1 m and 0-3 m. Sums for each borehole are listed with * indicating soils with a buried horizon. Bolded sums at bottom are means with standard error for all soils, only soils with a buried horizon and only soils without a buried horizon. Calculations contain uncertainty due to inherent uncertainties when using the van Bemmelen factor to estimate SOM and the equations of Saxton and Rawls to calculate bulk density.

Borehole#	Sum of C (Mg/ha) 0-1m	Sum of C (Mg/ha) 0-3m	Sum of N (Mg/ha) 0-1m	Sum of N (Mg/ha) 0-3m
1*	153	564	16.2	56.9
2	88.4	169	8.85	19.3
3*	114	270	11.2	25.3
4*	165	392	15.8	37.7
5	135	265	11.7	26.0
6*	150	312	13.7	27.9
7*	130	347	12.8	33.7
8	158	293	15.3	31.1
9	192	329	18.6	33.2
10	130	253	12.0	24.3
11	121	173	11.7	19.0
12	137	246	12.6	24.6
13*	164	321	15.0	27.0
14*	111	295	11.7	31.0
15*	108	278	10.9	27.1
16*	92.8	259	9.01	25.9
17*	110	283	12.4	28.7
18*	82.6	256	8.73	25.1
19*	105	308	9.96	30.3
20*	128	298	12.5	25.9
22*	145	253	13.1	24.0
26	104	147	11.4	19.8
27*	134	269	12.7	27.0
**Total Mean ± SE**	**128 ± 5.72**	**286 ± 17.2**	**12.5±0.511**	**28.3 ± 1.61**
**Buried Mean ± SE**	**126 ± 6.61**	**313 ± 20.4**	**12.4 ± 0.581**	**30.2 ± 2.12**
**Nonburied Mean ± SE**	**133 ± 11.3**	**234 ± 22.9**	**12.8 ± 1.04**	**24.7 ± 1.89**

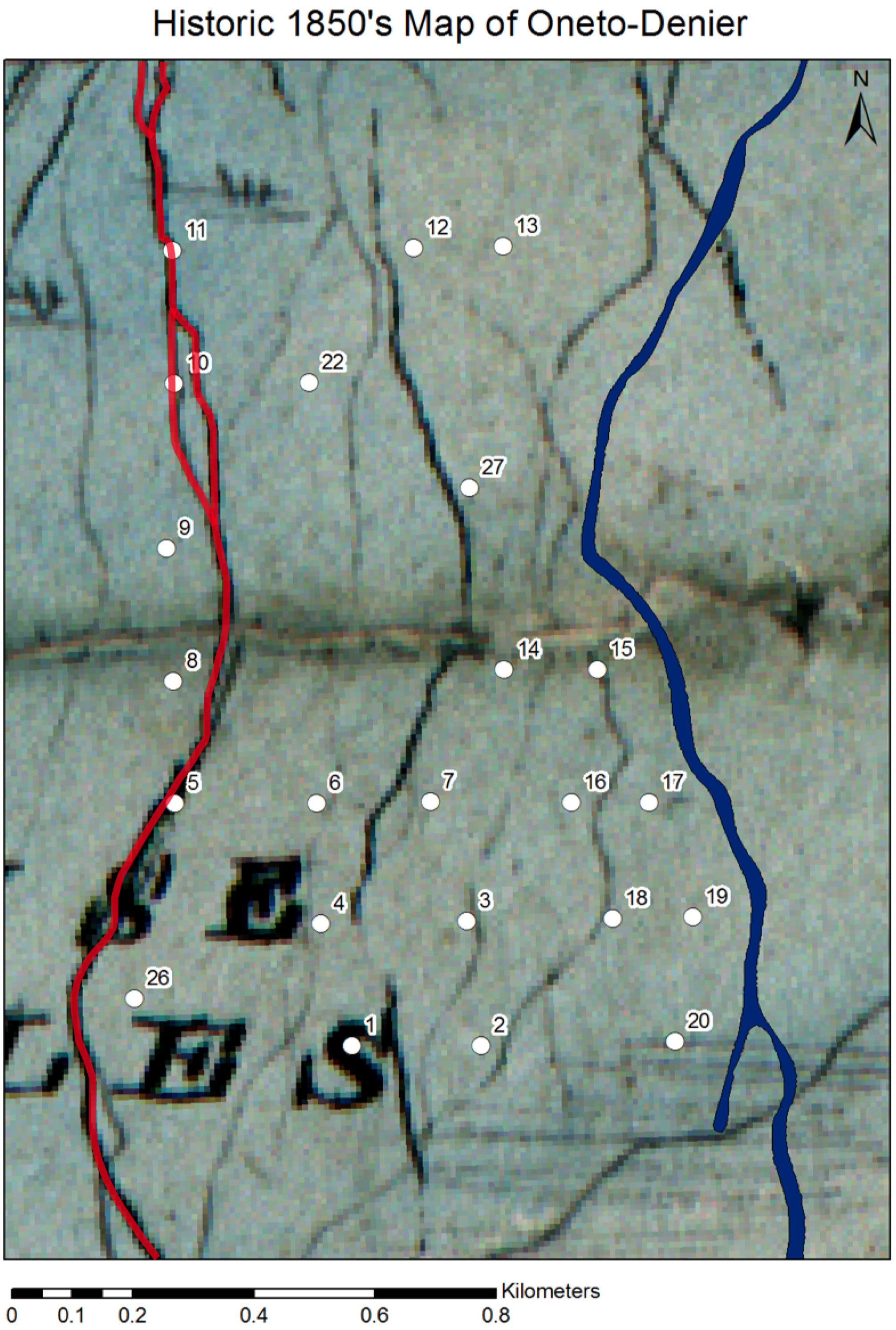

Supplementary Figure S1. Historic 1850's map of the Cosumnes field site before major human disturbance with the subsurface soil sample locations in white, the original Cosumnes River location highlighted in red, and the present day Cosumnes River location in blue created with ArcGIS version 10.4.1 (https://www.arcgis.com/features/index.html).

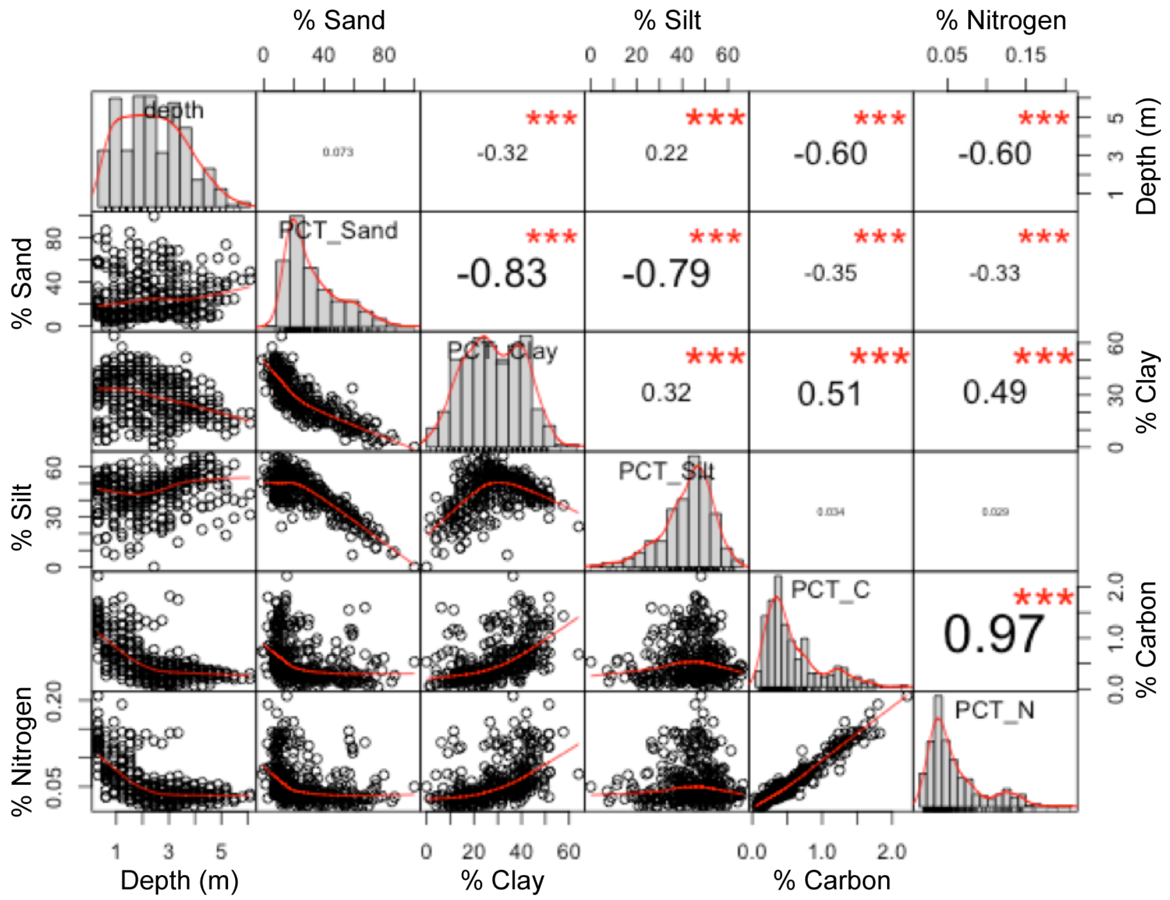

Supplementary Figure S2. Scatterplot matrix of particles size distribution and C and N content (PCT is %) from a depth of 0-3m. The frequency histograms in the diagonal show kernel density estimation overlays. The upper right triangle shows absolute Pearson correlations with significance asterisks (*0.05, **0.01, ***0.001). Font size indicates the strength of correlation. The lower left triangle below the diagonal shows the correlation scatterplots with a lowess smooth overlay. n=114

Supplementary Table S3. Buried soil horizon C contents and depths (bolded) compared to the overlying horizon (unbolded) including the percent increase in C at the buried horizon.

Borehole #	Depth (cm)	%C	% Increase
11	300-330	0.22	68
	**330-360**	**0.37**	
13	210-240	0.14	114
	**240-270**	**0.30**	
14	270-300	0.18	67
	**300-330**	**0.30**	
15	300-330	0.17	78
	**330-360**	**0.30**	
17	270-300	0.17	100
	**300-330**	**0.34**	
18	240-270	0.22	96
	**270-300**	**0.43**	
22	270-300	0.13	462
	**300-330**	**0.73**	

Supplementary Table S4. The range of global floodplain land cover and the first-order estimations of total SOC content for these global areas from 0-1 m and 0-3 m.

Floodplain Land Cover (ha)[11]	Total SOC Pg (0-1 m)	Total SOC Pg (0-3 m)
8×10^7	10.2	22.9
2×10^8	25.6	57.2

Supplementary Table S5. Equations used for bulk density calculation from Saxton and Rawls (2006)[28].

Variable	Equation	Eq. #
θ_{33t}	$\theta_{33t} = -0.251S + 0.195C + 0.11OM + 0.006(S \times OM) - 0.027(C \times OM) + 0.452(S \times C) + 0.299$	1
θ_{33}	$\theta_{33} = \theta_{33t} + [1.283(\theta_{33t})^2 - 0.374(\theta_{33t}) - 0.015]$	2
$\theta_{(S-33)t}$	$\theta_{(S-33)t} = 0.278S + 0.034C + 0.022OM - 0.018(S \times OM) - 0.027(C \times OM) - 0.584(S \times C) + 0.078$	3
$\theta_{(S-33)}$	$\theta_{(S-33)} = \theta_{(S-33)t} + (0.636\ \theta_{(S-33)t} - 0.107)$	4
θ_S	$\theta_S = \theta_{33} + \theta_{(S-33)} - 0.097S + 0.043$	5
ρ_N	$\rho_N = (1 - \theta_S)2.65$	6
ρ_{DF}	$\rho_{DF} = \rho_N \times DF$	7
α	$\alpha = \rho_{DF} / 2.65$	8
R_v	$R_v = (\alpha R_w) / [1 - R_w (1 - \alpha)]$	9
ρ_B	$\rho_B = \rho_N (1 - R_v) + (R_v \times 2.65)$	10

Supplementary Table S6. Symbols and their definitions used in equations of Supplementary Table 4 [28].

Symbol	Definition
S	Sand, %weight
C	Clay, %weight
OM	Organic Matter, %weight
θ_{33t}	33 kPa moisture, first solution, %volume
θ_{33}	33 kPa moisture, normal density, %volume,
$\theta_{(S-33)t}$	Saturated 33 kPa moisture, first solution, %volume
$\theta_{(S-33)}$	Saturated 33 kPa moisture, normal density, %volume
θ_S	Saturated moisture (0 kPa), normal density, % volume
ρ_N	Normal density, g cm^{-3}
DF	Density adjustment factor (0.9-1.3)
ρ_{DF}	Adjusted density, g cm^{-3}
α	Matric soil density/gravel density = ρ_{DF} /2.65 g cm^{-3}
R_w	Weight fraction of gravel (decimal), g g^{-1}
R_v	Volume fraction of gravel (decimal), g cm^{-3}
ρ_B	Bulk soil density (matric plus gravel), g cm^{-3}
